# Preliminary Assessment of Trace Metal Pollution and Their Bioaccumulation in Mollusks Inhabiting the Intertidal Sediments of the Atlantic Coast of Accra, Ghana

**DOI:** 10.1155/2022/9723272

**Published:** 2022-02-01

**Authors:** Emmanuel R. Blankson, Thyra F. Addison, Daniel Oduro, Juliet Ewool, Francis Gbogbo

**Affiliations:** Department of Animal Biology and Conservation Science, School of Biological Sciences, University of Ghana, Accra, Ghana

## Abstract

The present study investigated the trace metal levels in sediments, enrichment in sediments, and its bioaccumulation in mollusks collected from the intertidal sediments of the coast of Accra, Ghana, which is influenced by anthropogenic activities. The metals (Fe, Mn, Cu, Cr, and Zn) were analyzed in sediments. The metals (Mn, Cu, Cr, and Zn) were analyzed in the soft tissue of the mollusks collected from the different coastal areas. The results indicate that although Accra is the capital city and the coast receives tons of anthropogenic wastes, trace metal levels were low and there was no enrichment for the metals analyzed except Cu which showed minor to moderate enrichment in the coastal sediments. There was bioaccumulation of the trace metals in the soft tissue of the mollusks, and the results suggest that the mollusks studied, the gastropods (*Agaronia razetoi)* and the bivalves (*Tivela tripla*), can be used for biomonitoring.

## 1. Introduction

Trace metal pollution remains an environmental concern around the world. This is because metals are persistent in the environment and can cause toxic effects on organisms including humans [1–3]. Metals occur naturally in the environment, but their levels can be increased through anthropogenic sources such as mining, informal e-waste recycling, sewage treatment processes, and automobile emissions [4–6]. From these anthropogenic sources, most trace metals are transported by rivers, streams, and atmospheric deposition to marine ecosystems where they end up in the sediment by binding to suspended solids [7]. The extent of accumulation of metals in sediments and their bioavailability to aquatic organisms depends on the metal and sediment characteristics such as the organic content, particle size, pH, presence of iron and manganese oxides, pore-water ligands, and redox potential. [8–11].

Metals in sediment bioaccumulate in benthic invertebrates and present a potential health risk when they are fed on by organisms at higher trophic levels. As a result of the potential risk of metal exposure to organisms, metals are monitored by determining metal levels in sediment, water, and in the tissues of aquatic organisms such as fish, mollusk, and other invertebrates [12].

Generally, benthic macroinvertebrates are used for biomonitoring to assess the extent of environmental pollution. However, the species selected for biomonitoring is very important, and in the present study, we used mollusks because they are abundant with restricted movement to a particular local environment [12, 13]. They are also good bioindicators of environmental contamination because they bioaccumulate contaminants [12]. Among the mollusks, bivalves are preferred for biomonitoring, but previous studies have shown that gastropods can also be used for biomonitoring in aquatic environments [12, 14].

Accra, like most major cities in West Africa, is along the coast of the Gulf of Guinea. Accra is characterized by high population growth, urbanization, informal e-waste recycling, and industrial activities which contribute to the trace metal load along the coast of Accra. Previous studies have investigated metals in fish, bivalves, and gastropods [15, 16], and other studies have investigated metals in sediment or seawater from the coastal environment of Ghana [17–19]. However, studies that have investigated how metal concentrations in sediments influence their bioaccumulation in benthic invertebrates along the coast of Ghana and the Gulf of Guinea are rare. Therefore, in the present study, we investigated the extent of trace metal pollution along the Accra coast of the Gulf of Guinea, enrichment in sediments, bioaccumulation in mollusks, and the potential use of mollusks for biomonitoring. The importance of such findings in understanding metal pollution and bioaccumulation trends of trace metals in mollusks along the coast of the Gulf of Guinea compared to other coastal regions of the world cannot be neglected.

## 2. Materials and Methods

### 2.1. Study Area

The Accra coast is part of the Gulf of Guinea which is a large marine ecosystem on the Atlantic coast of West Africa. The Accra coast is the coastline of Accra, the capital city of Ghana which has lots of industries and is characterized by rapid urbanization. The coastline is approximately 40 km long, and it has a high population density, which is characterized by various economic activities such as fishing and recreation [20, 21]. The Accra coast has a tropical climate, and it is characterized by two wet seasons. These are between April and July and another in September–November that influence sediment transport along the coast [20]. The coastline is connected to lagoons through which streams and rivers discharge their content to the sea. The content includes domestic and industrial wastes which contribute to anthropogenic pollution along the coast of Accra, Ghana.

### 2.2. Field Sampling

**Figure 1:**
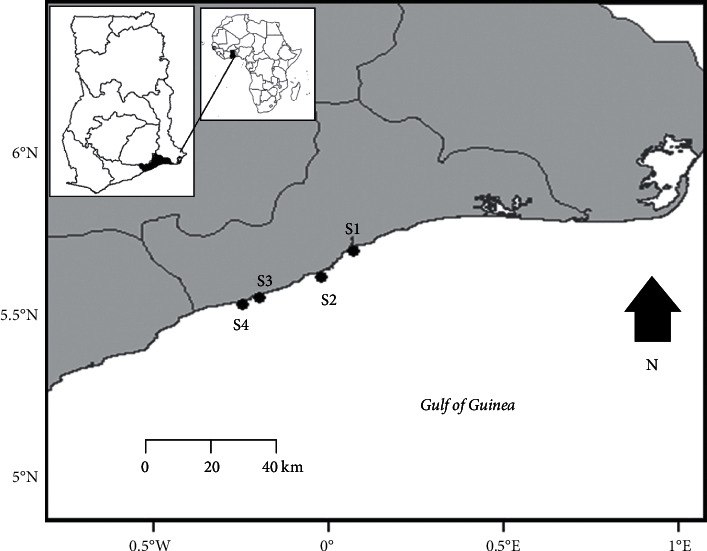
Map of Accra coast showing the sampling sites. S1, Sakumono coast; S2, Teshie coast; S3, Korle Gonno coast; S4, Jamestown coast.

The sediment samples were collected from intertidal sediments of four coastal areas along the Accra coast which are highly populated and influenced by anthropogenic activities such as urbanization and industrialization. These are Sakumono (S1), Teshie (S2), Korle Gonno (S3), and Jamestown (S4) coasts and are referred to as Site 1, Site 2, Site 3, and Site 4, respectively (Figure 1). Sakumono coast (S1) is close to the city of Tema, which has a lot of industries and a port. It is about 14.5 km away from the area with many industries and 8 km from the port. The Teshie (S2), Korle Gonno (S3), and Jamestown (S4) coasts are about 16 km, 7.4 km, and 8 km, respectively, from the area in the city of Accra with many industries. At each of the coastal areas, we collected the sediment with a corer with an area of 0.011 m^2^ along a transect from three different sites at a depth of 15 cm in the intertidal area. The sediments collected from the different sites were placed in ziplock bags and transported to the laboratory for analysis of Fe, Mn, Cu, Zn, and Cr. At each of the coastal areas, mollusks in the coastal sediments were collected by hand from the sites and transported on ice to the laboratory where they were stored at −20°C until analysis. The same mollusk species were not present at all the sites; we, therefore, collected the mollusks that were present at the different sites. We collected 17 and 2 individuals of the gastropods *Agaronia razetoi* from Sites 1 and 2, respectively, while at Site 3, there was no *Agaronia razetoi*; therefore, we collected 15 individuals of the bivalve *Tivela tripla*. We however did not find any mollusk in the sediment at the time of sampling at Site 4.

### 2.3. Analysis

#### 2.3.1. Organic Matter and Silt/Clay Content

The sediment organic matter and silt/clay content were quantified for the sediments collected from the four coastal areas along the coast of Accra (Table 1). The silt/clay content was determined by sieving a wet subsample of sediment through a 63 *μ*m mesh sieve. The sediment samples that were sieved and the fraction that was retained on the mesh were then dried at 60°C for 48 h and weighed. The organic matter in sediment was quantified using the loss-on-ignition method where approximately 1 g of ground sediment dried at 60°C was placed in a crucible and heated in the muffle furnace at 550°C for 4 h [22].

#### 2.3.2. Trace Metal Analysis in Sediment Samples

The sediment subsamples from the cores were dried in an oven at 60°C for 48 h in opened ziplock bags placed in acid washed beakers. The sediment was ground, and 10 ml of ternary mixture (HClO_4_: HNO_3_: H2SO_4_, 1 : 25 : 2.5) was added to 1 g of ground sediment in acid washed beakers and digested under a fume hood. After digestion, the samples were filtered into a 100 ml volumetric flask using a Whatman No. 42 filter paper. Distilled water was then added to the filtrate to make it to the 100 ml mark and analyzed with flame atomic absorption spectrophotometry (AAS) on PerkinElmer PinAAcle 900T.

#### 2.3.3. Trace Metal Analysis in Tissue Samples

The soft tissues of mollusks were removed from their shells using stainless steel scalpel blades. The soft tissues were placed in acid washed beakers and dried in an oven at 60°C to a constant weight. After quantification of the dry weight, 10 ml of ternary mixture (HClO_4_: HNO_3_: H2SO_4_, 1 : 25 : 2.5) was added to the tissues and digested under a fume hood. After digestion, the samples were filtered into a 100 ml volumetric flask using a Whatman No. 42 filter paper. Distilled water was then added to the filtrate to make it to the 100 ml mark and analyzed with flame atomic absorption spectrophotometry (AAS) on PerkinElmer PinAAcle 900T.

#### 2.3.4. Biota-Sediment Accumulation Factor (BSAF)

The BSAF provides a quantification of metal uptake by benthic organisms from the sediment. The BSAF was quantified as the ratio of metal concentrations in the mollusk and the sediments (with both concentrations expressed on a dry weight basis).

#### 2.3.5. Enrichment Factor

The anthropogenic input of metals in sediment was assessed using the enrichment factor. The enrichment factor was quantified by normalizing metal concentrations in samples and background concentration to a reference metal (Fe) concentration [23].

The enrichment factor was quantified by the following equation:

(1) $$\begin{array}{c} \text{EF}=\frac{\left(\text{CM}/\text{CFe}\right)\text{sample}}{\left(\text{CM}/\text{CFe}\right)\text{background}}, \end{array}$$

where CM is the concentration of the metal and CFe is the concentration of the reference metal. When EF < 1 shows no enrichment, EF < 3 shows minor enrichment, EF = 3–5 shows moderate enrichment, EF = 5–20 shows significant enrichment, EF = 20–40 shows very severe enrichment, and EF > 40 shows extremely high enrichment.

#### 2.3.6. Toxicity Assessment of the Trace Metals in Sediment

To assess the toxicity of trace metals to benthic organisms, we compared the concentration of each metal with the threshold effect levels (TEL). The TEL is a sediment quality guideline (SQG), and metals are considered to be toxic when they exceed the TEL values [24]. We subsequently calculated the toxic units (TU) by dividing the concentration of each metal by the threshold effect levels (TEL). A toxic unit greater than one shows that the metal levels in the sediment are possibly toxic to the benthic organism in the sediment [11].

### 2.4. Statistical Analysis

The data were checked for normality using the Shapiro–Wilk test. The datasets that were not normal were transformed or analyzed using the Kruskal–Wallis test. A one-way analysis of variance (ANOVA) was used to evaluate differences in Fe and Zn in sediments from the different sites. A Kruskal–Wallis test was used to analyze the differences in Mn, Cu, and Cr in sediments from the different sites. A one-way analysis of variance (ANOVA) was used to analyze the differences in bioaccumulation and BSAF values of metals in the tissue of mollusks from the different sites. The Pearson correlation was used to analyze the association between trace metals in sediment and sediment organic matter and silt/clay content. All statistical analyses were conducted with *R* version 3.5.1 [25].

## 3. Results

### 3.1. Trace Metals in Sediments

**Figure 2:**
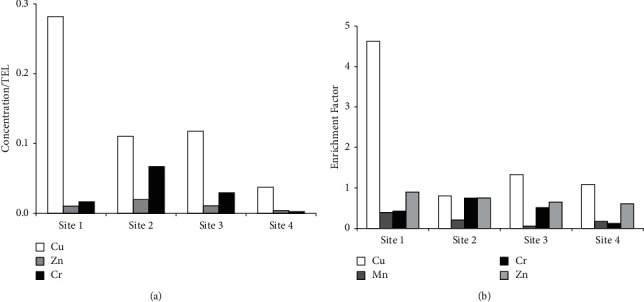
The toxic units (TU) of heavy metals (Zn, Cr, and Cu) concentration from the sampling sites (a) and the enrichment factor for metals (Mn, Zn, Cr, and Cu) in sediment collected from the sampling sites (b).

The average concentrations of metals in the sediments for all the sites are shown in Table 2. The Fe and Mn concentrations in the sediment on average were higher compared to all the other metals studied. Generally, the concentrations of all the metals were higher at Site 2 (Teshie coast) except for Cu which was higher at Site 1 (Sakumono coast). There were no significant (*P* > 0.05) differences in Cu, Mn, and Cr concentrations between the different sites. However, there were significant differences for Zn and Fe. The Zn concentrations in the sediment differed significantly (*P* < 0.05) between the sites. The Zn concentration was highest at Site 2 and lowest at Site 4. There was a fivefold difference between the highest and lowest concentration. The Fe concentrations in the sediment differed significantly (*P* < 0.05) between the sites. The Fe concentration was highest at Site 2 and lowest at Site 4. There was a fourfold difference between the highest and lowest concentration. Fe levels were significantly enhanced at Site 2 compared to the other sites. The toxic units (Figure 2(a)) were less than one for Cu, Zn, and Cr, thus suggesting no toxicity of these metals in the sediment.

### 3.2. Enrichment Factor

The result of the enrichment factors (Figure 2(b)**)** showed no enrichment of Mn, Cr, and Zn at all the sites. However, Cu showed minor to moderate enrichment at all the sites except for Site 2 (Teshie coast) where there was no enrichment. There was moderate enrichment at Site 1 and minor enrichments at Sites 3 and 4.

### 3.3. Relationship between Trace Metals and Sediment Properties

The sediment samples collected from the different sites had low organic matter and silt/clay content. The degree of OM in the coastal sediments from the different sites was in the order of Site 2 > Site 1 > Site 3 > Site 4. The degree of silt/clay content in the coastal sediments from the different sites was in the order of Site 2 > Site 4 > Site 3 > Site 1. The Pearson correlation between metals, organic matter, and silt/clay content was determined (Table 3). Fe in the sediment had a significant (*P* < 0.05) positive correlation with Zn and Cr. There was a significant positive correlation between Cu and Mn. There was also a significant positive correlation between Zn and Cr. The OM was significantly positively correlated to Fe and Cr.

### 3.4. Trace Metals in Mollusks

The average metal concentrations of mollusks sampled in the present study are shown in Table 4. There was no significant (*P* > 0.05) difference in Cu and Cr levels in the tissue of mollusks from the different sites. However, the Zn concentrations in the mollusk tissue differed significantly (*P* < 0.05) between sites. The highest Zn concentration was recorded in the mollusk from Site 2 (Teshie coast) and the lowest concentration in the mollusk from Site 3 (Jamestown coast). There was a fivefold difference between the highest and lowest concentrations which was significant. The Cr levels in the tissue of mollusks were low irrespective of the levels in the sediment suggesting low bioavailability of Cr to the mollusk. A comparison of the metal levels in the mollusk tissue in the present study to other studies [4, 6, 10, 26] shows that the metal levels in the mollusk along the Accra coast of the Gulf of Guinea are within recommended levels.

### 3.5. BSAF

The BSAF (Table 5) values for Cu differed significantly (*P* < 0.05) between the sites, and the highest BSAF value was for Site 2 (Teshie coast) and the lowest for Site 1 (Sakumono coast). The BSAF values for Zn differed significantly (*P* < 0.05) between the sites, and the highest BSAF value was for Site 1 and the lowest for Site 2. The BSAF values for Cr differed significantly (*P* < 0.05) between the sites. Cr generally had the lowest BSAF values, and the highest value was for Site 1 and the lowest for Site 2. Generally, for the metals studied, there was a trend where BSAF values were generally lower for the mollusk in sediment with higher metal concentrations.

## 4. Discussion

The concentrations of trace metals along the coast of Accra in the present study were low. However, the concentrations were within the range reported for other coastal areas along the coast of Ghana [16, 17]. The findings of the present study agree with a previous study [19], which showed that, along the Ghana coast, most trace metal concentrations were low in shallow areas of the continental shelf. A comparison of the metal levels in the present study to what has been reported along the coast in other parts of the world shows that the Accra coast is not polluted with Cr, Cu, Mn, Zn, and Fe [10, 11, 26, 27]. The trace metal levels along the Accra coast were within the recommended range and were below threshold levels in coastal and marine environments [24].

Although Accra is the capital city of Ghana and the coast receives tons of domestic and industrial wastes, the results of the enrichment factors suggest low anthropogenic input of trace metals. For all the metals studied except Cu, our results suggest that they are mainly from natural sources. This is because the results of the enrichment factors showed no enrichment of Mn, Cr, and Zn at all the sites. However, Cu showed minor to moderate enrichment at all the sites except for Site 2 (Teshie coast) where there was no enrichment. Site 1 (Sakumono coast) which had moderate enrichment of Cu in the sediment is close to Tema, a city in Accra that has a lot of industries and a port, and these may be an anthropogenic source of Cu to the marine environment. The positive correlations between Fe, Zn, and Cr and between Cu and Mn suggest that these metals are transported bonded to Fe or Mn oxides/hydroxides and are from a common anthropogenic and geologic origin [23, 28]. The evaluation of possible toxic effects of these metals by estimation of the toxic units suggests no toxicity of these metals as toxic units were less than one [11, 29, 30].

The bioaccumulation of metals in the mollusk was not significantly different between the sites for all the metals except Zn. Previous studies have shown that the difference in metal levels in the tissue of mollusks may be due to the concentration of metals, the environment, metabolism and the uptake routes such as diet, or from the ambient environment [9]. In the present study, the mollusk collected from Site 2 (Teshie coast) bioaccumulated more Zn. The difference may be due to the Zn levels in the sediments and organic matter. This is because sediment in Site 2 on average had more Zn levels and organic matter compared to the other sites. The bioaccumulation of Cu in the mollusk may have also been influenced by sediment organic matter. This is because although Cu levels in sediments from Site 2 were lower than those of Site 1, the average Cu levels bioaccumulated were higher in the mollusk from Site 2 than in the mollusk from Site 1. The Cr levels in the mollusk from the different sites were generally low, and Cr levels in the tissue relative to what is in the sediment suggest low bioavailability to the mollusk. This observation agrees with other studies which showed that mollusks do not bioaccumulate Cr readily from coastal sediments [9, 10, 31]. Mn, although it was detected in the sediments, was below the detection limit in the mollusks' soft tissues. The reason for this observation may be due to very low bioavailability to soft tissues of the mollusk. The other reason may be due to more bioaccumulation of Mn in the shell of the mollusks. It has been reported in other studies that Mn levels may occur at higher concentrations in the shell of some species of mollusks than in the soft tissue [32, 33]. However, in the present study, we did not quantify trace metals in the shells of the mollusks. Trace metal bioaccumulation has been reported in shells of mollusks [32, 33] and may influence trace metal levels in mollusks' soft tissues. The results suggest that the mollusks (*Agaronia razetoi* and *Tivela tripla*) studied in the present study may not be a good bioindicator for Cr and Mn pollution. The Cr, Zn, and Cu levels bioaccumulated by the mollusk in the present study compared to other studies around the world indicate that the levels in the tissue are low and within the recommended range [4, 10].

In the present study, bioaccumulation was quantified in two species of mollusks (*Agaronia razetoi* and *Tivela tripla).* Previous studies have shown that *Tivela tripla* may be a likely prey of *Agaronia razetoi* [34]. Therefore, there is a potential for more heavy metal bioaccumulation in *Agaronia razetoi* than *Tivela tripla* because of biomagnification of metals at higher trophic levels. However, the results of the present study suggest that bioaccumulation of metals was not influenced by the trophic levels but by the metal and the organic matter in the coastal sediment. In this study, the major challenge associated with the analysis of bioaccumulation of metals in mollusks from the various sites was the unavailability of the same species of mollusks in the spatial areas studied. The availability of the same species of mollusks in the various sites studied would have made the comparison stronger.

The BSAF values in the present study tend to be lower for the mollusk in sediment with higher metal concentrations. The trend where BSAF values tend to be lower for benthic organisms in more contaminated sediment has been reported in other studies [9, 22, 35]. The BSAF values for Zn, Cu, and Cr across the different sites ranged from 1.38 to 9.15, 0.87 to 3.41, and 0.07 to 0.39, respectively. The BSAF values for Zn, Cu, and Cr in the present study are similar to what has been reported in other studies for these metals in coastal areas around the world [6, 10, 36].

The organic matter in the coastal sediments also influenced the metal distribution in the sediment along the coast and in mollusks. The effect of the silt/clay content was not evident in this study. The sediment from Site 2 which had higher OM content had higher metal levels in the sediments compared to the other sites except for Cu which due to anthropogenic input was higher at Site 1. Trace metal bioaccumulation was higher in the mollusk from Site 2 except Cr which generally had low bioavailability and was not efficiently bioaccumulated by the mollusk. This observation agrees with previous studies which have shown that variation in metal levels in the sediment along the coast and in the mollusk is influenced by organic matter [9, 10, 28]. The organic matter in the sediment binds to the metals, decreases their release from the sediments, and thus increases metal levels in the sediment [9]. When the metals are retained in the sediment, this may increase their bioavailability to benthic organisms including mollusks through their diet or from their ambient environment. Some studies have shown increased bioaccumulation in bivalves because of increased organic matter in sediment [10].

## 5. Conclusion

The study shows that the trace metal levels in the coastal sediments of Accra and in the mollusks were below threshold levels. However, heavy metal levels in sediments except Cu tend to be higher in sediments with higher organic matter. The findings of this study also suggest that Cu levels in the sediment have an anthropogenic input. This is because the enrichment factor shows that Cu levels in the coastal sediments of Accra showed minimal to moderate enrichment. However, there was no enrichment for the other metals at all the other sites. There was bioaccumulation of Zn, Cu, and Cr in the mollusks; however, the concentration of Cr in the tissue relative to what is in the sediment suggests low bioavailability to the mollusks. Bioaccumulation of the heavy metals in the mollusk suggests that the gastropod (*Agaronia razetoi*) and the bivalve (*Tivela tripla*) can be used for biomonitoring of heavy metals.

## Figures and Tables

**Table 1:** The sediment organic matter (OM) and silt/clay (SC) content from the four sampling sites along the coast of Accra.

Sites	Organic matter (%)	Silt/clay content (%)
Site 1 (S1)	0.96 ± 0.27	0.05 ± 0.01
Site 2 (S2)	1.49 ± 0.26	0.24 ± 0.08
Site 3 (S3)	0.94 ± 0.10	0.16 ± 0.04
Site 4 (S4)	0.73 ± 0.16	0.21 ± 0.13

The values shown are mean ± SE (*n* = 3).

**Table 2:** Heavy metal concentrations (*μ*g/g) in sediments from the sampling sites.

Sites	Mn	Cu	Zn	Cr	Fe
Site 1 (S1)	7.76 ± 4.93 a	5.27 ± 3.62 a	1.30 ± 0.21 a,b	0.87 ± 0.32 a	1167.33 ± 184.85 a
Site 2 (S2)	9.3 ± 6.0 a	2.07 ± 0.33 a	2.47 ± 0.58 a	3.51 ± 1.52 a	2642.06 ± 460.21 b
Site 3 (S3)	1.63 ± 0.78 a	2.20 ± 0.7 a	1.37 ± 0.41 a,b	1.55 ± 0.29 a	1694.17 ± 186.50 a,b
Site 4 (S4)	1.93 ± 1.23 a	0.70 ± 0.25 a	0.50 ± 0.15 b	0.14 ± 0.13 a	659.42 ± 32.99 a

The values shown are mean ± SE (*n* = 3).

**Table 3:** Pearson correlation coefficient between metal concentrations, organic matter, and silt/clay content in the sediment.

	Mn	Cu	Zn	Cr	Fe	OM	SC
Mn	1						
Cu	0.58^*∗*^	1					
Zn	0.46	0.30	1				
Cr	0.55	−0.02	0.76^*∗*^	1			
Fe	0.11	−0.11	0.79^*∗*^	0.77^*∗*^	1		
OM	0.30	−0.25	0.55	0.86^*∗*^	0.67^*∗*^	1	
SC	0.24	−0.23	0.19	0.47	0.16	0.45	1

OM, organic matter; SC, silt/clay content.  ^*∗*^Significant correlation (*P* < 0.05).

**Table 4:** Heavy metal concentrations (*μ*g/g) in the tissue of the gastropod *Agaronia razetoi* (Ar) and bivalve *Tivela tripla* (Tt) from the sampling sites.

Sites	Cu	Mn	Zn	Cr
Site 1 (S1)^Ar^	4.575 ± 1.33 a	bd	11.9 ± 3.42 a,b	0.35 ± 0.01 a
Site 2 (S2)^Ar^	7.05 ± 0.25 a	bd	16.3 ± 1.30 a	0.28 ± 0.06 a
Site 3 (S3)^Tt^	4 ± 0.73 a	bd	3.15 ± 0.93 b	0.32 ± 0.03 a

The values shown are mean ± SE (with *n* = 4, 2, 4) from S1, S2, and S3, respectively. bd, below detection limit.

**Table 5:** Biota-sediment bioaccumulation factors of the gastropod *Agaronia razetoi* (Ar) and bivalve *Tivela tripla* (Tt) from the sampling sites.

Sites	Cu	Mn	Zn	Cr
Site 1 (S1)^Ar^	0.868 ± 0.25 a	bd	9.15 ± 2.63 a	0.39 ± 0.01 a
Site 2 (S2)^Ar^	3.41 ± 0.12 b	bd	1.38 ± 0.04 b	0.07 ± 0.02 b
Site 3 (S3)^Tt^	1.82 ± 0.33 a	bd	2.30 ± 0.68 b	0.20 ± 0.02 c

The values shown are mean ± SE (with *n* = 4, 2, 4) from S1, S2, and S3, respectively. bd, below detection limit.

## Data Availability

Data, associated metadata, and calculation tools are available on request from the corresponding author (erblankson@gmail.com).
